# Lenalidomide monotherapy and in combination with cytarabine, daunorubicin and etoposide for high-risk myelodysplasia and acute myeloid leukaemia with chromosome 5 abnormalities^☆^

**DOI:** 10.1016/j.lrr.2013.07.003

**Published:** 2013-08-28

**Authors:** M. Dennis, D. Culligan, D. Karamitros, P. Vyas, P. Johnson, N.H. Russell, J. Cavenagh, A. Szubert, S. Hartley, J. Brown, D. Bowen

**Affiliations:** a Christie Hospital, Manchester, United Kingdom; b Aberdeen Royal Infirmary, Aberdeen, United Kingdom; c John Radcliffe Hospital, Oxford, United Kingdom; d Western General Hospital, Edinburgh, United Kingdom; e Nottingham University Hospital, Nottingham, United Kingdom; f St. Bartholomew's Hospital, London, United Kingdom; g Clinical Trials Research Unit, University of Leeds, Leeds, United Kingdom; h St. James's Institute of Oncology, Leeds, United Kingdom

**Keywords:** Myelodysplasia, Acute myeloid leukaemia, Chromosome 5, Lenalidomide, Chemotherapy

## Abstract

Patients with high risk myelodysplasia (HR-MDS) and acute myeloid leukaemia (AML) with chromosomal changes involving deletion of the long arm of chromosome 5 (del5q), especially with complex karyotype, rarely have a durable response to combination chemotherapy. In the subgroup with monosomal karyotype there are no long term survivors (Fang et al., 2011) [1]. Recent experience indicates that the incidence of del5q in AML is ~20–30%, with only 20–25% of patients achieving complete remission (CR) (Farag et al., 2006) [2]. Additionally, therapy has significant toxicity, with induction death rates ~20% even in younger patients (Juliusson et al., 2009) [3]. This lack of efficacy provides the clinical rationale for combination/sequential therapy with Lenalidomide and combination chemotherapy. Dose dependent haematological toxicity is the major safety concern with such a combination protocol. Therefore we conducted a phase 2 study, AML Len5 (ISRCTN58492795), to assess safety, tolerability and efficacy of lenalidomide monotherapy, followed by lenalidomide with intensive chemotherapy in patients with primary/relapsed/refractory high risk MDS or AML with abnormalities of chromosome 5.

Patients with high risk myelodysplasia (HR-MDS) and acute myeloid leukaemia (AML) with chromosomal changes involving deletion of the long arm of chromosome 5 (del5q), especially with complex karyotype, rarely have a durable response to combination chemotherapy. In the subgroup with monosomal karyotype there are no long term survivors [1]. Recent experience indicates that the incidence of del5q in AML is ~20–30%, with only 20–25% of patients achieving complete remission (CR) [2]. Additionally, therapy has significant toxicity, with induction death rates ~20% even in younger patients [3]. Conversely, patients with low risk MDS and del5q can respond dramatically to lenalidomide at conventional doses [4,5]. Thus, studies have investigated lenalidomide therapy in AML/HR-MDS. A phase 2 study of lenalidomide in HR- MDS with the del5q abnormality (alone or with other cytogenetic abnormalities) indicated a 20% CR rate with lenalidomide 10 mg once a day, escalated to 15 mg in suboptimally responding patients. However, all responders had isolated del5q [6]. Subsequent studies have evaluated escalating doses up to 50 mg daily but response rates remained ~4% [7]. A phase 1 study in relapsed/refractory AML patients also indicated that lenalidomide can be tolerated up to 50 mg daily, but there were no CR's in the del5q population. Those that did respond had low presenting white cell counts suggesting limited efficacy as monotherapy in proliferative disease [8] Additionally, the Nordic group had similar findings when they assessed lenalidomide monotherapy up to 20 mg daily in a phase 2 study for MDS/AML patients with any form of chromosome 5 abnormality and noted no response in patients with TP53 mutation [9]. More recently a phase 1 study of sequential therapy with azacitidine and lenalidomide has identified an alternative approach capable of inducing haematological improvement and complete cytogenetic remissions, although has significant haematological toxicity [10].

This lack of efficacy provides the clinical rationale for combination/sequential therapy with Lenalidomide and combination chemotherapy. As already outlined dose dependent haematological toxicity is the major safety concern with such a combination protocol. Therefore we conducted a phase 2 study, AML Len5 (ISRCTN58492795), to assess safety, tolerability and efficacy of lenalidomide monotherapy, followed by lenalidomide with intensive chemotherapy in patients with primary/relapsed/refractory high risk MDS or AML with abnormalities of chromosome 5.

Patients were considered eligible if they had a diagnosis of primary/relapsed/refractory AML (as defined by WHO 2008) or high risk MDS (defined as IPSS INT-2/High) with chromosome 5 cytogenetic abnormalities (including del5q) and were suitable for intensive chemotherapy. Cytogenetic analysis was undertaken at regional specialist cytogenetic laboratories using conventional chromosome G-banding on bone marrow cultures. Specific FISH analysis for del5 q was not undertaken.

The main exclusion criteria were prior use of lenalidomide or other investigational agents within the last 4 weeks. Composite primary endpoint was early death rate (defined as death within 30 days of starting combination therapy) and survival with platelet recovery (>100×10^9^/l) 42 days after the last dose of course one combination therapy. If treatment was found to be safe and acceptable, the CR rate (CR/CR with incomplete haematopoietic recovery) was assessed at day 21 post the last cycle of combination chemotherapy, and used to determine the sample size for the study. A four-stage phase II non randomised trial design (Sargent) was used to incorporate the possibility that the trial might be inconclusive based on the observed CR rate. Stopping rules were included at four time points; after 10, 19, 30 and all 39 patients had completed the first course of combination chemotherapy. For patients recruited with ≥5% blasts, further stopping rules were specified as follows for lenalidomide monotherapy: more than 20% having a treatment-related death; more than 30% withdrawn due to delayed recovery of blood counts with hypoplastic bone marrow.

All patients were recruited and consented from 6 UK leukaemia centres. The treatment schedule was designed such that all patients would receive an initial cycle of lenalidomide monotherapy 10 mg daily on days 1–21 of a 28-day cycle. Remission status was then assessed from a day 28 bone marrow.

Responses were defined as; CR <5% blasts in the bone marrow and haematopoietic recovery. CR with incomplete haematopoietic recovery (CRi) fulfilling all criteria for CR except for haematopoietic recovery.

Partial Response (PR) ≥5% blasts in the bone marrow but blasts having reduced by ≥50% from baseline. No Response (NR) <50% blast reduction in the bone marrow from baseline.

Patients who had presented with HR-MDS (< 5% blasts) and had no blast excess at day 28 and who also achieved haematopoietic recovery, received further 28 day cycles of lenalidomide monotherapy 10 mg daily on days 1–21. After receiving 3 cycles of lenalidomide monotherapy, patients in remission were considered for allogeneic stem cell transplant. If transplant were not an option then patients would continue with maintenance lenalidomide.

Patients who presented with HR-MDS or AML (≥5% blasts) who at day 28 had achieved a PR could receive a second cycle of lenalidomide monotherapy. If CR or no response at day 28, then patients progressed to combination chemotherapy with Lenalidomide administered at 10 mg once daily for 10 days concurrently with ADE (10+3+5) induction therapy [Cytarabine 100 mg/m^2^ twice daily I.V. for 10 days, Daunorubicin 50 mg/m^2^ days 1,3, and 5 I.V. and Etoposide 100 mg/m^2^ daily days 1–5 I.V.]. If CR/PR was achieved it was consolidated with a further course of combined lenalidomide and ADE (8+3+5), followed by a course of combined lenalidomide and high dose Cytarabine1.5 g/m^2^ twice daily by intravenous infusion on days 1, 3 and 5 (6 doses). Patients in remission were considered for allogeneic stem cell transplant.

Between August 2009 and May 2010, 14 patients were recruited, median age 66 (range 40–75). The majority, 10 (71%) had AML; four (29%) had HR-MDS. The disease was classified as; primary (9–64%), relapsed (3–21%) and refractory (2–14%). Only two (14%) had an isolated del5q, whilst 12 (86%) had additional chromosomal abnormalities. All 14 patients received lenalidomide monotherapy. One patient with HR-MDS had an initial PR then subsequent CR and continues in CR on maintenance lenalidomide, 21 months since therapy initiation. Nine patients did not respond and went on to have the combination therapy. Therefore, following monotherapy, no patients with blasts ≥5% achieved a remission. Four patients discontinued therapy, including three attributable to death whilst receiving monotherapy; all 3 had ≥5% blasts at diagnosis. The overall early death rate was 21%, and 27% in patients recruited with ≥5% blasts. The patient flow is summarised in Fig. 1.

Of the nine patients who received combination therapy, three achieved CR/CRi (33%) and one patient achieved PR (11%), for an ORR of 44%. (summarised in Table 1).

Two patients who achieved CR underwent allogeneic transplantation. One died of an infection-related death in CR 16 months post transplant; the other relapsed 17 months post transplant but remains alive. Use of lenalidomide in close proximity to allogeneic transplant conditioning has been linked to increased incidence of acute GVHD [11]. However, this was not evident in the 2 patients reported here. The other patient who achieved CRi, failed to ever achieve sufficient haematological recovery to enable further therapy- eventually relapsing 301days following the achievement of CRi prior to death. The patient who achieved PR died from neutropenic infection, prior to being able to receive further therapy.

Adverse events were frequent in this challenging cohort of patients. In total 9 SAEs were reported during combination therapy, 6 of which were suspected to be related to the therapy and included neutropenic sepsis or thrombocytopenia. All patients experienced the anticipated haematological toxicity. Other notable toxicities included grade 3 ALT rise (22%) and venous thrombo-embolism (11%). This is summarised in greater detail in Table 2.

Blood product utilisation was in keeping with the recognised requirements for such patients undergoing intensive chemotherapy. During the first cycle of combination chemotherapy, patients received a median of 10 units of blood (range 4–24) and 10 units of platelets (range 2–29). Intravenous antibiotic usage was for a median duration of 21 days s (range 13–44). The median duration of hospital in-patient stay was 32 nights (range 16–44).

Seven patients were evaluable for stem/progenitor cell immunophenotyping at diagnosis using previously published protocols [12] (Fig. 2). 4/7 presented with an abnormally expanded haemopoietic stem cell (HSC) population (Lin-CD34+CD38-CD90+CD45RA-). 2/7 presented with an expanded LMPP (Lin-CD34+CD38-CD90-CD45RA+) and GMP (Lin-CD34+CD38+CD123+CD45RA+) compartment. 1/7 presented with expanded MPP (Lin-CD34+CD38-CD90-CD45RA-) and CMP (Lin-CD34+CD38+CD123+/lowCD45RA-) populations. Thus, there was heterogeneous expansion of populations with leukaemic stem cells (LSC) activity.

Sequential bone marrow samples were available for 6/7 patients. The 2/7 with an expanded HSC compartment at diagnosis did not respond to combination therapy and there was no significant reduction in the size of the abnormally expanded immunophenotypic HSC compartment. Upon achievement of remission expanded GMP and LMPP populations were demonstrated in the patient who responded to lenalidomide monotherapy and continues in CR on maintenance lenalidomide. Conversely, however, the patient who presented with low percentage of blasts, achieved CR and remained relapse free for more than a year, continued to have an expanded HSC compartment. Of the two patients with an expanded GMP/LMPP compartment, one did not respond to therapy and continued to have abnormally expanded LMPP and GMP populations after combination therapy. The other had an abnormally high percentage of progenitor populations (MPP/GMP) at a point when he achieved CR by morphological criteria. Thus, we were unable to identify a consistent stem/progenitor profile at diagnosis, or following response to therapy, in this heterogeneous patient cohort.

It is clear that our data confirm previous findings that lenalidomide monotherapy at a dose of 10 mg daily is ineffective as induction therapy in HR-MDS/AML patients with increased marrow blasts. Indeed, the consequent delay in administering cytotoxic chemotherapy appears detrimental to disease control and patient outcome. In view of the unacceptable early death rate the trial was halted for consideration of a trial protocol amendment.

An independent Data Monitoring & Ethics Committee (DMEC) carried out a simultaneous review of data for the 9 patients who had received combination chemotherapy. Although there were no early deaths with the combination therapy, 7 patients had failed to achieve platelet recovery. The formal stopping rule for the first 10 patients was either ≥5 early deaths or ≥8 patients not recovering platelets and surviving. One further patient received only a single dose of ADE and did not receive the lenalidomide, as the patient was nil by mouth. As recommended by the DMEC, this patient was not considered as receiving combination therapy. Note also that this patient did not recover their platelets and survive (died two days after the ADE because of a SAE suspected to be related to monotherapy). Therefore, the formal stopping rule would have been activated if this patient had been considered as receiving combination therapy.

Patient recruitment had been challenging and well behind the predicted rate with patients in the UK entering competing studies due to the absence of immediate cytogenetic analysis at presentation with AML. It was, therefore, concluded that it would not be possible to complete the study within the available time. A decision was therefore made by the trial management group to permanently halt recruitment rather than amend the protocol. Although the combination therapy was deliverable, emerging experience suggests that alternative combination strategies may deliver similar activity with attenuated toxicity. One such example is the combination of lenalidomide with azacitidine, for which there are now a number of similar phase I/II studies ongoing and the final results are awaited [13–15].

In conclusion, lenalidomide monotherapy at a dose of 10 mg daily is ineffective as induction therapy in HR-MDS/AML patients with increased marrow blasts. When lenalidomide is combined with ADE chemotherapy there is predictable and manageable toxicity, which is not clearly greater than with combination chemotherapy alone. Efficacy is limited in this particularly adverse patient cohort. Currently very few of these patients, even after allogeneic transplant, achieve long-term disease free survival such that innovative combination strategies are urgently required.

## Disclosure

M. Dennis has received research funding and honoraria from Celgene. D. Culligan has received honoraria from Celgene. D. Karamitros has nothing to disclose. P. Vyas, P. Johnson, NH Russell, J. Cavenagh, A. Szubert, S. Hartley and J. Brown have nothing to disclose. D. Bowen has received honoraria from Celgene.

## Figures and Tables

**Fig. 1:**
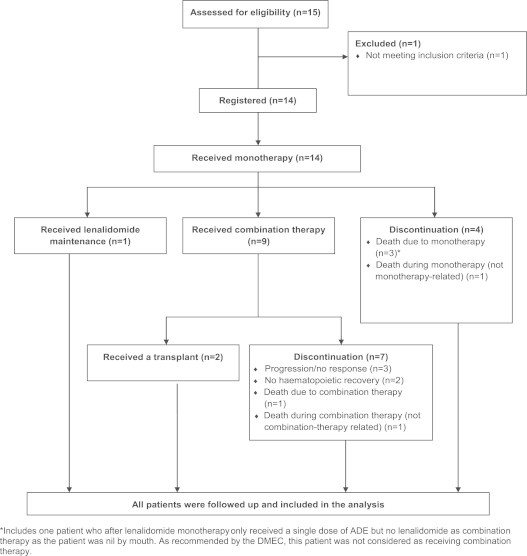
^*^Includes one patient who after lenalidomide monotherapy only received a single dose of ADE but no lenalidomide as combination therapy as the patient was nil by mouth. As recommended by the DMEC, this patient was not considered as receiving combination therapy.

**Fig. 2:**
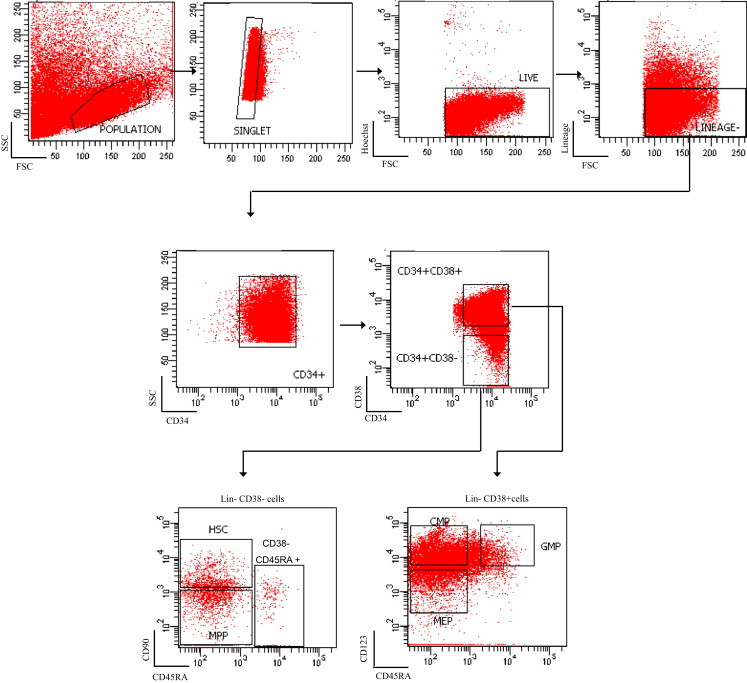
Immunophenotyping of haematopoietic stem and progenitor populations of patients with high-risk MDS and AML with chromosome 5 abnormalities. CD34+ or total MNCs were analysed for the expression of lineage markers, CD34, CD38, CD45RA, CD90 and CD123. The gating strategy is shown. HSC: Lin-CD34+CD38-CD90+CD45RA-, MPPs: Lin-CD34+CD38-CD90-CD45RA-, LMPP-like: Lin-CD34+CD38-CD90-CD45RA+. CMPs:LinCD34+CD38+CD123+/lowCD45RA. GMPs:LinCD34+CD38+CD123+CD45RA+ and MEPs: Lin-CD34+CD38+CD123-CD45RA-.

**Table 1:** Response 21 days post last cycle of combination therapy.

**Table 2:** Adverse reactions during combination chemotherapy.
